# Application of New Triple Hook-Shaped Conformal Cooling Channels for Cores and Sliders in Injection Molding to Reduce Residual Stress and Warping in Complex Plastic Optical Parts

**DOI:** 10.3390/polym13172944

**Published:** 2021-08-31

**Authors:** Abelardo Torres-Alba, Jorge Manuel Mercado-Colmenero, Juan de Dios Caballero-Garcia, Cristina Martin-Doñate

**Affiliations:** Department of Engineering Graphics, Design and Projects, Campus Las Lagunillas s/n., University of Jaen, Building A3-210, 23071 Jaen, Spain; ata00001@red.ujaen.es (A.T.-A.); jmercado@ujaen.es (J.M.M.-C.); jdcg0004@red.ujaen.es (J.d.D.C.-G.)

**Keywords:** conformal cooling, sustainability, injection molding, industrial design, manufacturing, numerical simulation

## Abstract

The paper presents a new design of a triple hook-shaped conformal cooling channels for application in optical parts of great thickness, deep cores, and high dimensional and optical requirements. In these cases, the small dimensions of the core and the high requirements regarding warping and residual stresses prevent the use of traditional and standard conformal cooling channels. The research combines the use of a new triple hook-shaped conformal cooling system with the use of three independent conformal cooling sub-systems adapted to the complex geometric conditions of the sliders that completely surround the optical part under study. Finally, the new proposed conformal cooling design is complemented with a small insert manufactured with a new Fastcool material located in the internal area of the optical part beside the optical facets. A transient numerical analysis validates the set of improvements of the new proposed conformal cooling system presented. The results show an upgrade in thermal efficiency of 267.10% in comparison with the traditional solution. The increase in uniformity in the temperature gradient of the surface of the plastic part causes an enhancement in the field of displacement and in the map of residual stresses reducing the total maximum displacements by 36.343% and the Von—Mises maximum residual stress by 69.280% in comparison with the results obtained for the traditional cooling system. Additionally, the new design of cooling presented in this paper reduces the cycle time of the plastic part under study by 32.61%, compared to the traditional cooling geometry. This fact causes a very high economic and energy saving in line with the sustainability of a green mold. The improvement obtained in the technological parameters will make it possible to achieve the optical and functional requirements established for the correct operation of complex optical parts, where it is not possible to use traditional cooling channels or standard conformal cooling layouts.

## 1. Introduction

The use of precision polymer optics is becoming an increasing necessity today as products demand sophisticated light handling components to achieve desired results [1,2]. Even if the optical properties of materials such as glass are very stable, their manufacturing process is especially complex considering the time limitations and requirements demanded in the industry. Nevertheless, optical pieces manufactured in plastic are sturdy and low-cost, produced in one only step despite the geometric complexity. These reasons make plastic optical parts crucial for contemporary industrial development [3,4,5].

The diversity of optical products for the automotive sector caused the development of geometries requiring high-thickness ratios. In the injection molding process, the thickest area of the part presents heat accumulation because of slower cooling. This fact produces not only a slower cooling time but also an uneven shrinkage [6,7,8] that generates warping, affecting the optical properties of the plastic part [9].

Compared to the most stringent technical parts, which require tolerances of tenths of a millimeter, the requirements for optical components are up to 100 times higher, and not only with regard to a certain dimension, but also along its entire surface thus guaranteeing its correct operation. A designed geometry with a wide variation in the thickness ratios can originate thermally induced residual stress during the injection cooling process [10]. Residual stress results in a slight local reduction of the optical properties, and therefore, also of the transmitted light [11].

Achieving an even distribution of temperatures to eliminate residual stress using conventional cooling channels is very difficult. In this line, conformal cooling channels have greater flexibility to adapt their geometry to the complex topological requirements of the plastic optical part [12,13]. The use of conformal cooling channels improves the uniformity in the cavity surface temperature reducing in that way the residual stress thermally induced throughout the cooling process [14,15]. Additive technology enables the manufacturing of complex conformal cooling channels to best match the shape of the cavity and core in the mold. In this way, a uniform cooling in areas where heat can be difficult to trap, like hardly accessible and high thickness areas, can be achieved. Conformal cooling channels allow uniform heat dissipation for optical parts as well as high cooling efficiency [16].

Although some authors have made use of the advantages of employing conformal channels for cooling pieces of diverse geometry, only a few have studied their application for cooling optical parts with complex geometries. These works, mainly focus on the cooling of optical lenses with complex geometry [17]. Chung [17] combines the analysis of finite elements with an algorithm based on gradients and a robust genetic algorithm to obtain the optimal design of the cooling channels for an optical lens. According to his research, conformal channels can reduce the temperature differences on the mold surface, as well as the ejection time, and the warping.

The design of molds for complex optical parts requires a great number of mobile devices in order to manufacture all the topological part features. In complex cases, mobile devices are responsible for molding a large part of the geometry of the piece, limiting the space of the cavity plate [18]. This fact has a great influence on the design of the cooling layout since the design of the cavity cooling is performed entirely on the side cores. Side cores usually present complex geometry and reduced dimensions as they have to adapt to the geometry of the part. This precludes the design of a continuous cooling layout using straight or standard conformal channels that completely surround the part [19,20,21]. Likewise, the small dimensions of mobile devices limit the use of large diameter cooling channels dividing channels into individual zones adapted to the requirements of each mobile device. This fact greatly hinders the use of traditional channels since they require compliance with the high design and sizing requirements of the CNC manufacturing process.

The high optical requirements force the design of optical parts of great depth, which causes deep cores in the mold. These areas are highly difficult to cool, forcing the use of baffles normally far from the internal surface of the part due to compliance with the manufacturing criteria of the CNC process. The use of baffles in optical pieces with deep cores prevents the correct thermal exchange between coolant flow and plastic melting [22,23].

Conformal cooling channels provide greater design flexibility by reducing the distance to the part, presenting more functional layouts for parts with complex geometries [24,25,26]. However, the use of standard conformal channels also presents limitations in cases with a lack of accessibility and space for cooling. These boundary conditions force the use of conformal channels with very small diameters, which can cause insufficient heat exchange and obstructions due to interference from foreign objects.

The use of inserts manufactured with Fastcool [27] material is presented as an innovative solution to deal with the lack of space in very deep and difficult access regions. Nevertheless, the high conductivity of Fastcool inserts requires the use of specific layouts for their cooling, since the tool steel of the mold is not able to evacuate the heat produced by the insert quickly enough to generate a uniformity of temperatures, achieving an efficient cycle time. In this case, conformal cooling layouts are considered a feasible option to evacuate the heat generated by these inserts in those areas of the mold with a lack of accessibility and where the design requirements prevent the direct use of standard conformal channels.

To solve the problems proposed, the paper presents a new design of a triple hook-shaped conformal cooling channels for application in optical parts of great thickness, deep cores and high dimensional and optical requirements. In these cases, the small dimensions of the core and the high requirements regarding warping and residual stresses prevent the use of traditional and standard conformal cooling channels. The research combines the use of a new triple hook-shaped conformal cooling system with the use of three independent conformal cooling sub-systems adapted to the complex geometric conditions of the sliders that completely surround the optical part under study. Finally, the new proposed conformal cooling design is complemented with a small insert manufactured with a new Fastcool material located in the internal area of the optical part beside the optical facets.

In this way, it is possible to reduce warping and residual stresses in the manufacture of highly complex optical parts with high thickness ratios, meeting the demanding requirements of the automotive industry. In parallel, a reduction in the production cycle time and energy consumption of the mold is achieved, producing a sustainable mold in line with the demands of the current environment. Additionally, the uniformity in the temperatures of the surface of the piece is improved by eliminating hot spots and differential shrinkage. The paper exceeds the state of the art, being capable of cooling optical parts with deep cores, high thickness and small details, being very useful in an area as important and widespread worldwide as is the molding manufacturing of automotive optical parts.

## 2. Materials and Methods

### 2.1. Analysis and Geometrical Features for the Plastic Part Manufactured through the Injection Molding Process

In this item, the topological and technological features related to the plastic part under study are depicted. Specialized insights about the selection of the plastic material for the injection molding manufacturing process, boundary conditions, and topological features are likewise determined.

**Figure 1:**
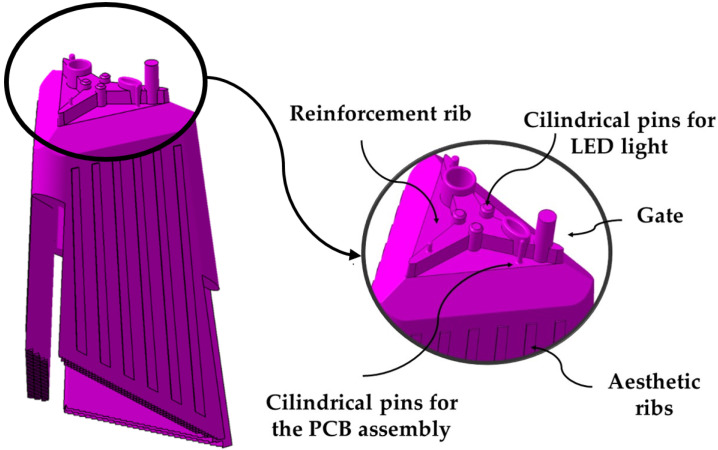
Optimal part under study and topological details of its geometry.

The geometry presented in the paper is a plastic optical piece, whose main function is to guide and control the luminous flux from various LED points, distributing it evenly over the illumination plane. The piece (see Figure 1), presents a complex geometric design with great influence on its manufacturing process. The piece has small dimensions with a bounding box of (67 × 52 × 127) mm and a thickness of 6 mm. The upper part of the part is characterized by including a set of 7 lugs or pins whose main function is to serve as an injection point for the part and as a means of connection to the LED optical devices and the optical control PCB. The set of pins is attached to three reinforcing ribs distributed equidistantly, forming an angle of 120° with each other. The function of the ribs is to reinforce the base of the pins against possible stresses to which the part will be subjected throughout its useful life, as well as, to reduce the effect of the plastic material shear at the injection gate. Figure 1 shows a picture of the optical piece under study as well as the topological details that characterize its geometry.

**Figure 2:**
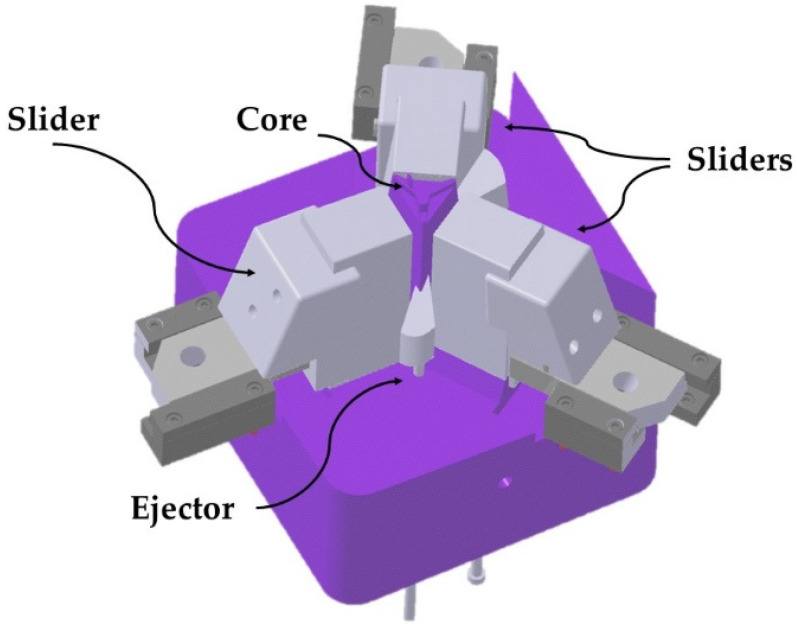
Side sliders.

The base of the optical piece presents an irregular geometry caused by the different heights at which the three lateral surfaces that characterize the piece are found. Likewise, the lower surface of the piece is characterized by including a set of optical facets in charge of the distribution and channeling of the LED light. The lateral surfaces include a set of 23 faceted grooves of thickness 0.3 mm of an aesthetic character whose function is to avoid that the surfaces present a completely smooth appearance. The manufacture of the lateral grooves requires the use of three sliders in the injection mold since the geometries are not demoldables. The sliders of the mold completely surround the contour of the optical piece (see Figure 2).

**Figure 3:**
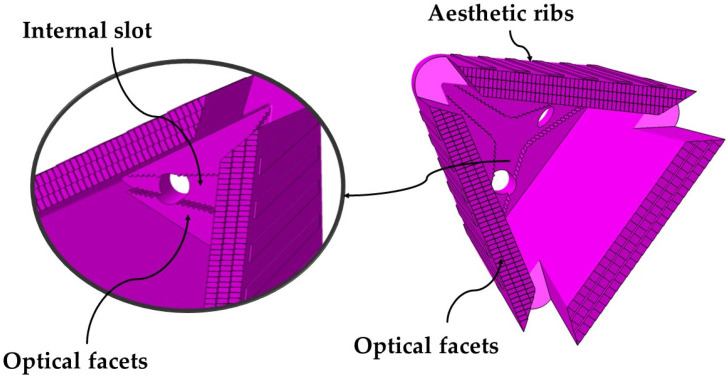
Geometric details of the internal area of the optical piece.

The optical piece is characterized by the inclusion of a deep concave geometry area inside, which makes that the core plate presents a great depth that is difficult to cool. Additionally, the upper internal area of the part, close to the injection point, includes a set of faceted optical grooves. These facets require precise molding which forces to design a cooling in the core plate that avoids possible warping that could invalidate the optical function of the piece. Figure 3 presents a picture of the internal area of the optical piece under study as well as the details of the optical facets included in the upper internal surface.

The dimensions of the core of the mold in contact with the internal area of the piece prevent the use of ejector pins to extract it from the mold. For this reason, the design of the mold requires the use of three external ejector inserts located in three lateral recesses of the optical piece analyzed. The different heights at which the lateral surfaces of the piece are located influences the parting line of the mold to be located in different planes.

**Figure 4:**
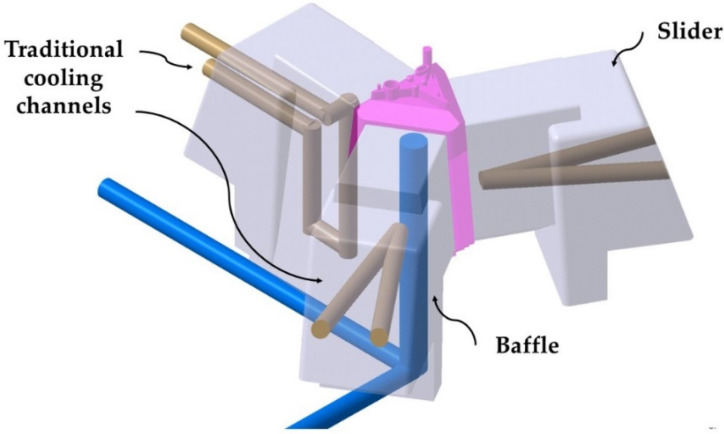
Current layout of the traditional cooling of the core and sliders of the mold.

The geometry of the plastic part greatly influences the design of the injection mold and fundamentally the design of its cooling system. Figure 4 shows the diagram of the cooling of the optical part with the current cooling layout of the mold, using traditional methods for its manufacture. The mold makes use of a cooling based on the design of 8 mm diameter straight cooling channels located in each of the sliders. It also uses baffle-type elements to cool the core of the mold. The small dimensions of the core and the side cores, as well as the fulfillment of the traditional manufacturing requirements that guarantee the structural safety of the mold, make the design of the cooling layout extremely difficult. Likewise, the great depth and small dimensions of the core, require the use of a single baffle with a diameter of 12 mm and a separation of 20 mm to cool the internal area of the piece. The cooling of the mold is complemented by two straight channels of 8 mm diameter in the upper part of the mold cavity in charge of cooling the material injection gate.

The cooling of deep cores in pieces of great thickness is highly complex since it requires cooling designs capable of evacuating the heat accumulated in specific areas of the core, and of avoiding differential shrinkage and subsequent warping in the molded plastic part. The optical piece analyzed in the paper has a slotted and faceted interior zone very close to the injection point (see Figure 3). This area, which is 6.5 mm thick and difficult to access, locally accumulates a large amount of heat, that must be removed by cooling. The use of standard conformal cooling systems is not valid for cooling small deep cores and thick molded parts. Additionally, the reduced dimensions of the core would require the use of small diameter standard conformal channels with little cooling path, preventing the correct thermal exchange between the coolant flow and the molten plastic.

To solve this problem, the paper presents a new design of triple hook-shaped conformal cooling for application in pieces of great thickness and deep cores. In these cases, the small dimensions of the core prevent the use of standard conformal cooling systems. Likewise, the research combines the use of the new triple hook-shaped conformal cooling system with the use of three independent conformal cooling channels included in the three small side cores. In this way, it is possible to adequately cool the sliders of the mold using three conformal cooling sub-systems adapted to the particular geometric conditions of each slider. Finally, the new proposed conformal cooling design is combined with a small Fastcool element located in the internal area of the piece where the optical facets are located.

**Figure 5:**
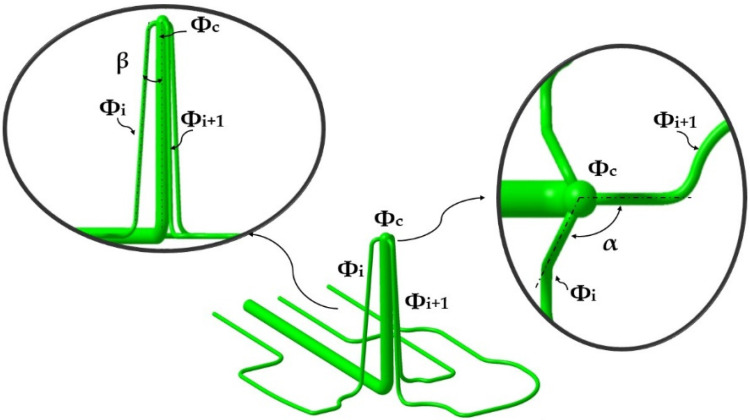
Conformal cooling triple hook-shaped channel.

The new triple hook-shaped conformal cooling design presented in this paper aims to cool the core of the mold in contact with the three side surfaces of the part, as well as to cool the Fastcool insert. The new conformal triple hook-shaped conformal design is made up of a central channel with a domed end from which three channels depart equally spaced at an angle α in the horizontal plane and an angle β of vertical slope. The triple hook design meets the design criteria in additive manufacturing [27] eliminating the use of supports in its manufacture. Equation (1) indicates the sizing criteria that the diameters of the triple hook- shaped conformal channels must meet, where $\phi_{c}$ is the diameter of the central channel and $\phi_{i}$ the diameter of the lateral channels. The central channel $\phi_{c}$ must not exceed the value of 10 mm to avoid material collapse when manufacturing the channel upper area. Figure 5 shows a picture of the new triple hook-shaped conformal channel design for cores presented in the paper.

(1) $$\phi_{c}=\overset{3}{\underset{i=1}{\sum }}\phi_{i}|\;\phi_{c}\leq 10\;\mathrm{mm}\;\;\phi_{i}=\phi_{i+1\;}$$

The upper part of the core close to the injection point of the part presents a hot spot that causes a great thermal imbalance in the cooling of the part. 

**Figure 6:**
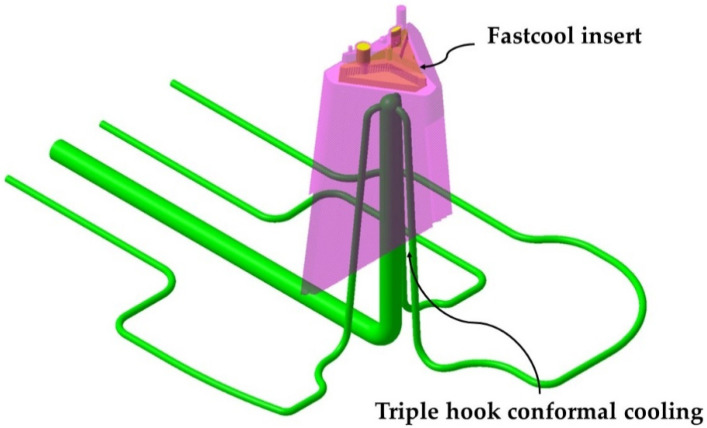
Fastcool insert.

To eliminate this thermal imbalance, the new triple hook conformal cooling design is complemented by the use of a small flat-shaped insert of a new Fastcool material [28] located on the upper inner surface of the core. In this way, it is possible to establish a complete cooling design adapted to the geometrical and functional requirements of the part, capable of extracting the heat accumulated in the gate area, eliminating possible warping in the upper optical zone. Figure 6 indicates the location of the Fastcool insert used to cool the area of the optical facets close to the injection point.

**Figure 7:**
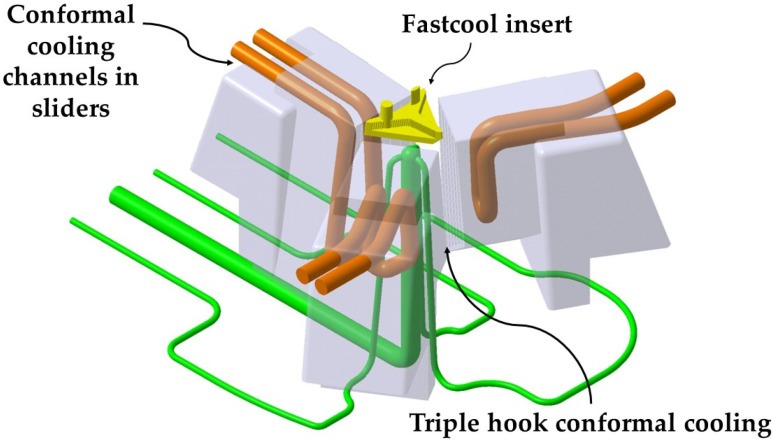
Conformal cooling of the core and sliders of the mold.

The area of the three slides in charge of molding the lateral surfaces of the part is cooled using three independent conformal cooling layouts adapted to the geometry of each slide. In this way, it is possible to cool complex parts whose mold cavities are formed mostly by small sliders and in which the standard conformal spiral or zigzag layouts that surround the part are impossible to use. Figure 7 presents a picture of the conformal cooling of the core and sliders of the mold presented in the paper, formed by the use of the new conformal cooling triple hook shaped combined with a small Fastcool flat insert for cooling the mold core.

**Figure 8:**
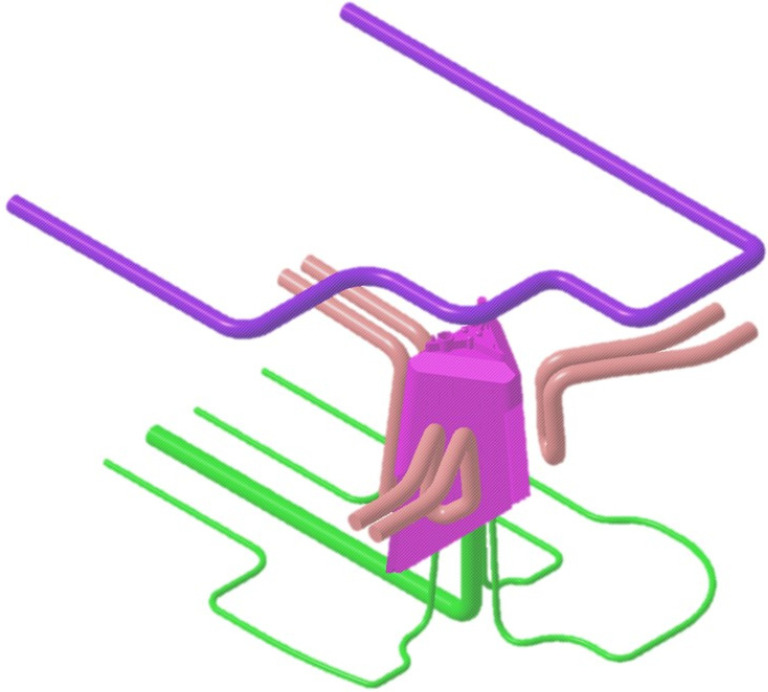
Complete cooling of the cavity, core and sliders of the injection mold por the presented optical part.

The mold cavity is cooled using three independent conformal cooling channels adapted to the geometric requirements of the three side sliders of the mold. Finally, the cooling of the mold cavity is complemented by two conformal cooling channels of 8 mm diameter in the upper part of the cavity plate in charge of cooling the material injection gate (see Figure 8). Table 1 indicates the dimensions of the main different elements used in the conformal cooling of the optical part.

### 2.2. Plastic Part Material

The optical part under study is manufactured with PC Lexan 121R plastic material from the company Sabic [29] obtained by chemical recycling. Therefore, the mechanical, thermal, and chemical properties of the original plastic are maintained without compromising the sustainability of the injection process. This thermoplastic material is a Polycarbonate that allows designers and manufacturers the facility for design freedom, aesthetics enhancements and cost reductions. Furthermore, due to its physical properties and specifications, it can be applied and used in optical plastic parts. The magnitudes of the main physic, mechanical and thermal properties of the material PC Lexan 121R are indicated in Table 2.

## 3. Implementation and Results

In this manuscript, two proposals for the design of the cooling system for the plastic part under study are presented and compared. On the one hand, the current traditional cooling system with perforated straight channels whose manufacture is carried out using traditional machining processes and tools. And, on the other hand, a new optimized cooling system that combines conformal triple hook-shaped cooling channels with a fastcool-type metal insert. So, the manufacture of this proposal is based mainly on the 3D additive manufacturing process using laser sintering (SLS). Additive manufacturing technologies are line with current sustainability requirements. The SLM additive manufacturing process allows the manufacture of conformal channels adapted to the free shape of the geometric surface of the plastic part [30,31]. The geometric CAD design of both configurations is performed using the Catia V5—6R2020 3D CAD geometric modeling software [32]. Likewise, to evaluate and analyze the thermal and technological parameters that define the cooling phase of the plastic part, numerical simulations of a thermal type are modeled using the numerical and commercial software Moldex3D R17-CoreTech System Taiwan, [33]. In this way, the results of the thermal and technological parameters obtained from both proposed cooling system configurations can be compared, establishing the one that optimizes and improves, on the one hand, the cooling phase of the plastic part, as well as the thermal efficiency. Both the 3D CAD modeling process and the numerical analysis of the different cooling system configurations proposed in this manuscript have been carried out using an MSI notebook with an Intel (R) Core- Intel corporation EEUU(TM) i-77700HQ CPU @ 2.80 GHz.

### Thermal Modeling of Numerical Simulations

**Figure 9:**
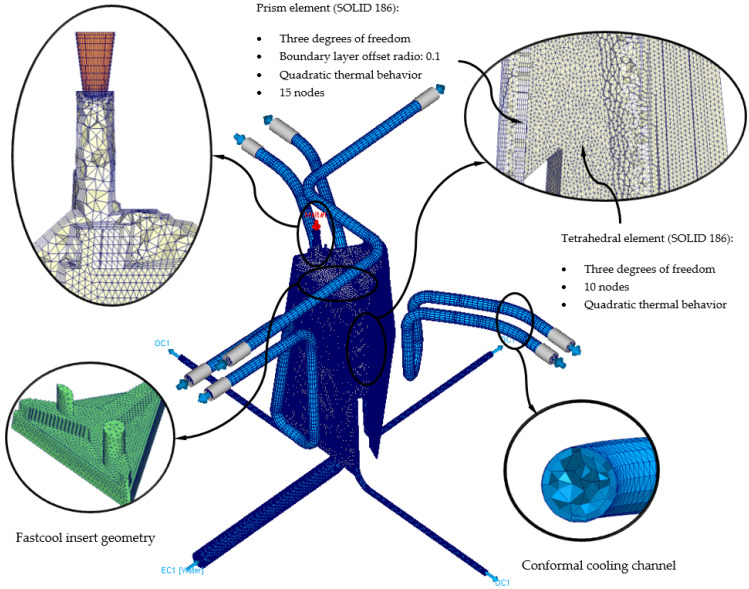
Mesh details for the new conformal cooling triple hook—shaped and Fastcool insert configuration.

**Figure 10:**
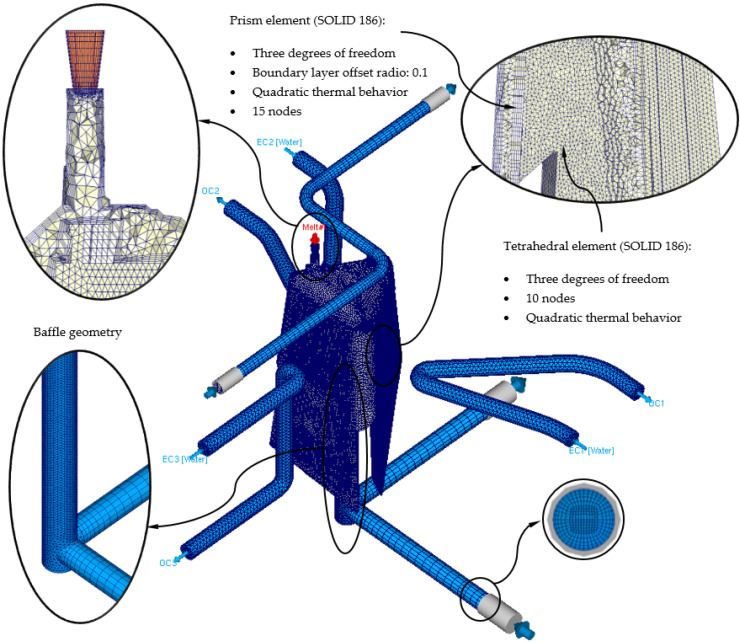
Mesh details for the traditional cooling configuration.

The definition of rheological and thermal simulations using CAE numerical software allows the analysis of the cooling phase of a plastic part and how the main elements of the injection mold and the most representative technological parameters of said phase influence and interact. Likewise, the results of the parameters obtained from the numerical simulations allow establishing whether the design of the main elements that make up the cooling system, meets the minimum industrial technical requirements that are established to validate the manufacture of the plastic part. In this section, the preprocessing configuration used for each of the different numerical simulations carried out is detailed. At the beginning of this preprocessing phase, the discretization of the different geometric elements to be analyzed numerically must be defined. That is, the three-dimensional meshes for the geometric elements of the injection mold must be defined, as well as the geometric parameters that define them. The commercial software Moldex 3D R17 [33] has a Moldex Designer meshing module, in which the geometric parameters of the meshes created can be configured and established. Table 3 shows the magnitude of the geometric parameters used during the meshing process, as well as their configuration. Said geometric parameters have been adjusted to the smallest and most relevant geometric detail or precision of the plastic part under study. However, the selection of the type of element used is important when carrying out the meshing process. In this way, three-dimensional elements of the second-order tetrahedron have been selected, called SOLID 186. These elements have 10 main nodes, located at the vertex of the tetrahedron, and 4 secondary nodes, located at the midpoint of the edges of the tetrahedron (see Figure 9). In addition, each of said nodes has 3 degrees of freedom in the main coordinate axes, notably improving the precision of the temperature field parameters and displacements in the solution obtained. Besides that, and in order to improve the precision of the numerical simulation, a series of three-dimensional elements of the prism type have been defined along the contact surfaces between the different elements that make up the injection mold. Elements are placed on the surface of the cooling channels or between the surface of the plastic part and the cavity and core surface of the injection mold cavity. These elements have 6 main nodes located at the vertices of the prism, and 9 secondary nodes located at the midpoint of the edges of the prism (see Figure 9). Furthermore, each of said nodes has 3 degrees of freedom in the main coordinate axes. The selection of this type of element is established by the “Boundary Layer Mesh” operation, which establishes a series of layers from the interface surfaces previously mentioned (see Figure 10). The average length of these elements is configured from an offset ratio or percentage of the size of the average element of the mesh. In this case, and as Table 3 shows, the offset ratio selected for the generation of the meshes is 0.1 and the number of Boundary Layers is equal to 5. The use of this meshing operation allows modeling with greater precision the roughness generated between the surface of the cooling channels and the coolant flow, as well as the layer of solidified plastic material that is generated when the molten plastic front comes into contact with the surfaces of the injection mold cavity.

**Figure 11:**
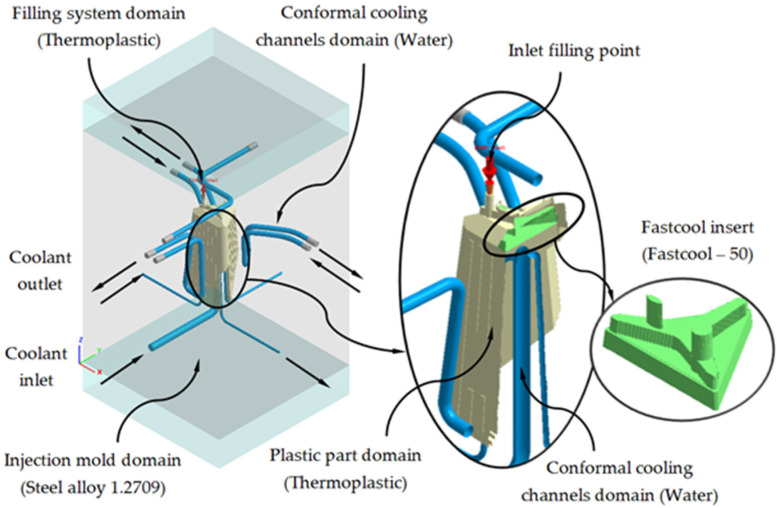
Materials and boundary conditions defined for the new conformal cooling triple hook—shaped and Fastcool insert configuration.

Next, we proceed to define the material assigned to each one of the elements of the injection mold and the plastic part, as well as the physical, thermal, and rheological properties of each one of them. As shown in Figure 11, the plastic part is manufactured from the thermoplastic material PC Lexan 121 R [28]. The main feed channel, from which the filling of the mold cavity begins, has associated, like the plastic part, the PC Lexan 121 R thermoplastic material. For the cooling channels, both for the traditional configuration and for the conformal configuration, the material water is defined as the coolant flow. For the geometry defined as injection mold, the assigned metal material is a P20 steel alloy, and finally, for the Fastcool insert, used in the conformal cooling system solution, the metallic material used is a Fastcool-50 steel alloy. Table 4 shows the magnitude of the physical, thermal and rheological properties defined in the numerical simulations for each material used. As can be seen, the use of a Fastcool insert, whose metallic material is Fastcool-50 [27], considerably improves the thermal properties of the metallic material of the mold. In this way, the area of the plastic part that is in contact with the Fastcool insert will present greater heat exchange and, therefore, will improve thermal efficiency throughout the cooling phase. Therefore, as shown in Figure 11, said Fastcool insert is located in the inner central core of the plastic part. Well, in this region a large amount of residual heat accumulates and presents greater difficulty to be uniformly re-cooled, with respect to the rest of the geometric regions of the plastic part.

Likewise, the definition of the thermoplastic material in the simulation software must be accompanied by numerical models that allow the modeling of both the behavior of the viscosity of the material and the behavior of its PVT curve. In addition, the manufacturer of the material [29] recommends the magnitude of a series of temperatures for each of the phases of the manufacturing cycle of the plastic part. Table 5 shows the parameters recommended by the manufacturer and the viscosity and PVT curve models of the thermoplastic material defined in the numerical simulation software.

**Figure 12:**
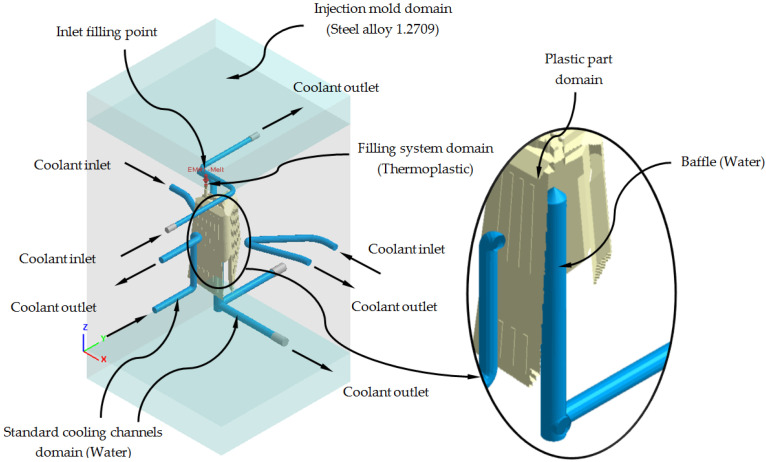
Materials and boundary conditions defined for the traditional cooling configuration.

As shown in Figure 11 and Figure 12, each numerical analysis carried out has associated a set of boundary conditions, which establish the technological parameters of pressure and initial temperature for the input of the molten plastic front to the injection mold cavity and the flow of the coolant flow to the cooling channels. For the input of the molten plastic front to the injection mold cavity, the upper surface of the main feed channel (see Figure 11) is established as a boundary condition, an injection temperature of 295 °C, and a maximum injection pressure of 160 MPa. For the cooling channels, firstly, both the inlet and outlet surfaces of the coolant flow are defined (see Figure 11); secondly, an initial temperature of the coolant flow of 80 °C is determined, and finally, a pressure magnitude that allows the front of the coolant flow to develop in turbulent regime. That is, the Reynolds number of the coolant flow along the cooling channels is greater than 1.5 × 10^4^. Likewise, and according to the recommended parameters offered by the manufacturer of the thermoplastic material, the initial temperature of the injection mold is set at 80 °C.

Table 6 shows the magnitude of the technological parameters used in the modeling of the filling and cooling phase of the numerical simulations carried out for the present case study.

To complete the definition of the preprocessing phase of the numerical simulations carried out, the following configurations relative to the solver used to solve the numerical models of the simulations carried out are defined:

- The analysis of the cooling phase of the plastic part is carried out in a transitory regime or “Cooling transient”. Given the defined cooling time (see Table 6), the solver analyzes the process and the evolution of the cooling of the plastic part over time. This type of analysis allows obtaining and saving solutions of the field of temperatures and displacements for different time intervals. The time interval defined in each numerical simulation carried out is 10 s.
- The modeling of the coolant flow along the cooling channels is done with the “Run 3D cooling channels” operation. This operation makes it possible to define a roughness magnitude on the surfaces of the cooling channels and improves the analysis of turbulence on their surface. The magnitude of the defined roughness is equal to 0.02 mm.
- The type of solver used is the maximum variation of mold temperature and its convergence criteria are temperature difference equal to 1 °C and maximum cycle number equal to 10 cycles.

After completing the definition of the pre-processing phase of the different numerical analyzes performed, the set of thermal and rheological results obtained is presented. From their analysis and evaluation, it is determined that the configuration of the triple hook-shaped conformal cooling channels for cores together with the use of conformal cooling channels adapted to the sliders and the Fastcool insert optimizes the cooling phase and improves the efficiency and thermal performance of the injection mold for the plastic part object of study.

**Figure 13:**
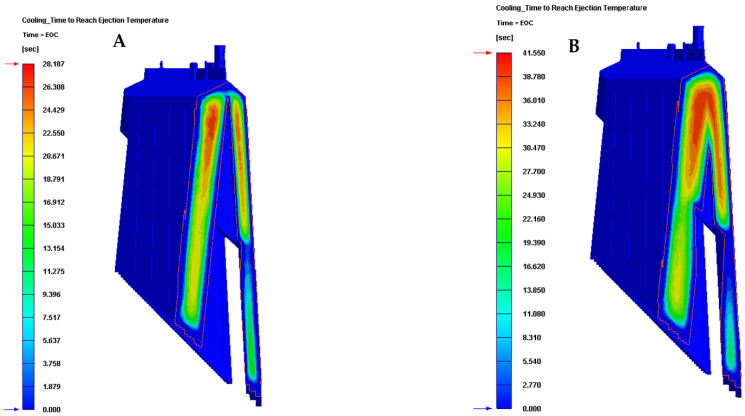
Time to reach ejection temperature (s). (**A**) Conformal cooling and fastcool insert. (**B**) Traditional cooling.

Firstly, Table 7 and Figure 13 show the results obtained for the parameter time to reach the ejection temperature of the plastic part for each of the cooling system configurations proposed in this manuscript.

In this way, the results presented in Table 7 and Figure 13 show that the new configuration of the conformal cooling system presented in this paper, together with the use of a Fastcool insert, improves the time until reaching the ejection temperature of the plastic part, with respect to the configuration of the current traditional cooling system. Therefore, it can be determined that the use of this new type of conformal cooling channels, accompanied by an insert with high thermal performance, reduces the cycle time of the plastic part under study by 13.363 s or by 32.161%, compared to the classical geometry and configuration of perforated straight cooling channels.

**Figure 14:**
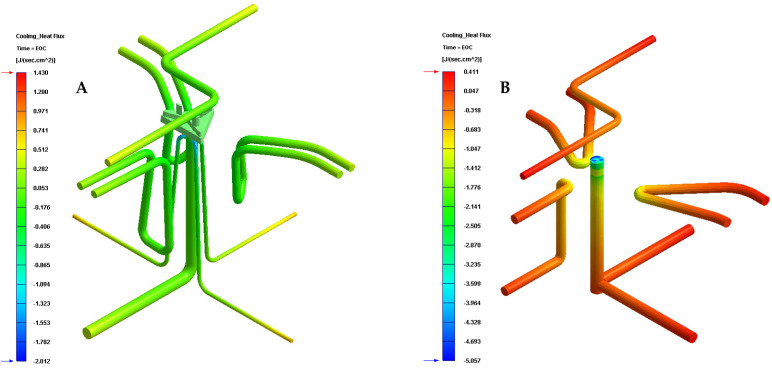
Heat flux (J/s·cm^2^). (**A**) New conformal cooling system and Fastcool insert. (**B**) Traditional cooling.

Table 8 and Figure 14 show the results obtained for the heat flow parameter that is exchanged between the cooling mechanisms and the plastic part, for each of the cooling system configurations proposed in this manuscript.

**Figure 15:**
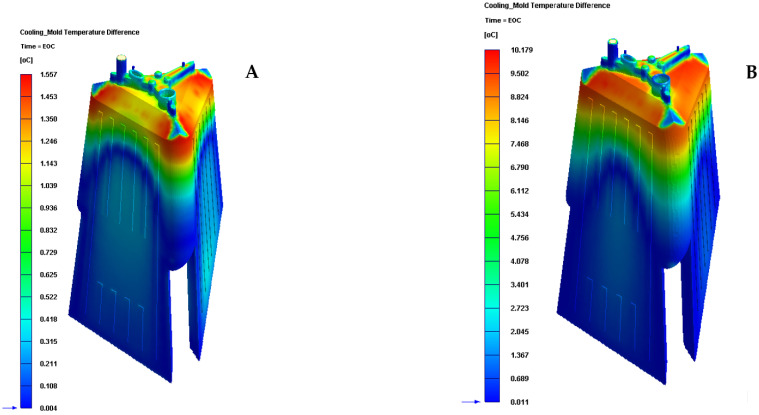
Cooling mold temperature difference (°C). (**A**) New conformal cooling system and Fastcool insert. (**B**) Traditional cooling.

Likewise, the results presented in Table 8 and Figure 14 show that the configuration of the new presented conformal cooling system, together with the use of a Fastcool insert, increases and optimizes the heat flow exchanged between the plastic part and the different cooling mechanisms defined for cooling the plastic part. In particular, the increase in heat exchange produced, compared to the standard cooling configuration, is 2.062 J/s·cm^2^, which translates into an improvement in thermal efficiency of 267.10%. Table 9 and Figure 15 show the results obtained for the temperature gradient along the surface of the plastic piece under study in this manuscript.

The results presented in Table 9 and Figure 15 show that the configuration of the new conformal cooling system, together with the use of a Fastcool insert, reduces the temperature gradient along the surface of the plastic part concerning the traditional cooling system configuration. In particular, this reduction represents an improvement of 84.701% in the uniformity of the temperature map throughout the plastic part under study. In addition, the new conformal cooling channel solution, together with the Fastcool insert, meets the industrial validation requirements of the manufacture of the plastic part since the magnitude of the temperature gradient on the surface of the plastic part is less than 10 °C. However, for the traditional cooling system, this condition is very close to being fulfilled.

**Figure 16:**
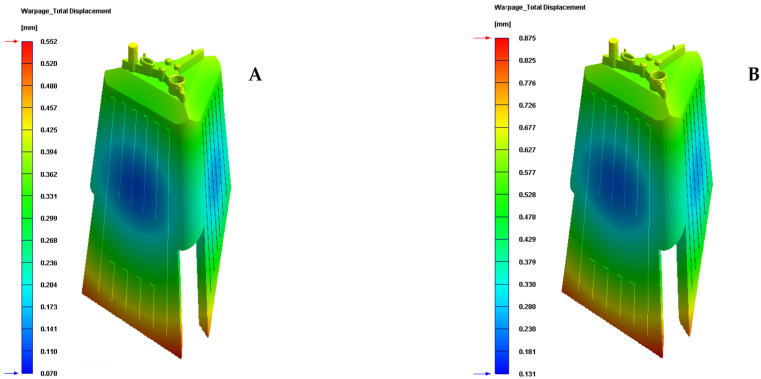
Warpage total displacement (mm). (**A**) New conformal cooling and Fastcool insert. (**B**) Traditional cooling.

**Figure 17:**
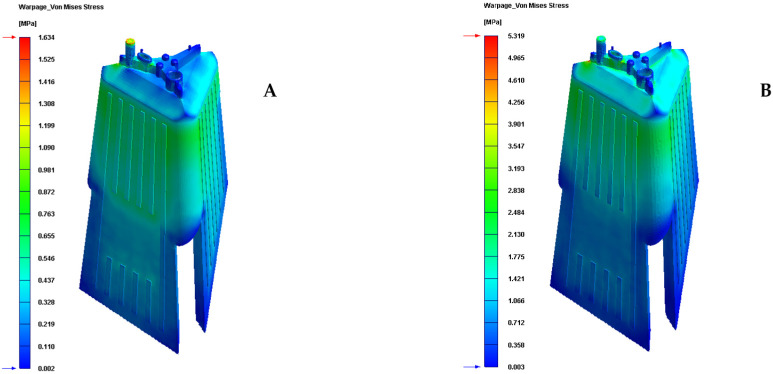
Warpage Von—Mises stress (MPa). (**A**) New conformal cooling system and Fastcool insert. (**B**) Traditional cooling.

Finally, given that the plastic part under study is an optical lighting part, it is important to analyze and check the field of displacements and the map of residual stresses resulting after the manufacturing process. Table 10 and Table 11 and Figure 16 and Figure 17 show the displacement field and the residual stress map of Von—Mises along with the geometry of the plastic part under study after the cooling phase.

As can be seen, for the new conformal cooling system with a Fastcool insert, the increase in uniformity in the temperature gradient of the surface of the plastic part causes a decrease and improvement in the field of displacement and the map of residual stresses on the plastic part. In particular, the total maximum displacements are reduced by 0.318 mm and the Von—Mises maximum residual stress by 3.685 MPa in comparison to the results obtained for the traditional cooling system. Likewise, the improvement obtained in these technological parameters makes it possible to achieve the optical and functional requirements established for the correct operation and validation of the plastic part under study in this manuscript.

## 4. Conclusions

The paper presents a new design of triple hook-shaped conformal cooling channels for application in optical parts of great thickness, deep cores and high dimensional and optical requirements. In these cases, the small dimensions of the core and the high requirements regarding warping and residual stresses prevent the use of traditional and standard conformal cooling channels. The research combines the use of a new triple hook-shaped conformal cooling system with the use of three independent conformal cooling sub-systems adapted to the complex geometric conditions of the sliders that surround completely the optical part under study. Finally, the new proposed conformal cooling design is completed with a small insert manufactured with a new Fastcool material and located in the internal area of the optical part where the optical facets are located. A transient numerical analysis validates the improvements of the new proposed conformal cooling system presented. The results show that the configuration of the new presented conformal cooling system, together with the use of a Fastcool insert, increases and optimizes the heat flow exchanged between the plastic part and the different cooling mechanisms defined for cooling the plastic optical part. In particular, the upgrade in heat exchange produced, compared to the traditional cooling configuration, is 2.062 J/s·cm^2^, which translates into an improvement in thermal efficiency of 267.10%. The enhancement in uniformity in the temperature gradient of the surface of the plastic part causes a decrease and improvement in the field of displacement and the map of residual stresses on the plastic part. In particular, the total maximum displacements are reduced by 0.318 mm or by 36.343% and the Von—Mises maximum residual stress by 3.685 MPa or by 69.280% in comparison to the results obtained for the traditional cooling system. Additionally, the new design of cooling presented in this paper reduces the cycle time of the plastic part under study by 13.363 s or by 32.161%, compared to the classical geometry and configuration of perforated straight cooling channels, which causes a very high economic and energy saving in line with the sustainability of the mold. The amellioration obtained in these technological parameters will make it possible to achieve the optical and functional requirements established for the correct operation and validation of complex optical parts, where it is not possible to use traditional cooling channels or standard conformal cooling layouts.

## Figures and Tables

**Table 1:** Geometric parameters used in the design of conformal cooling system.

Nomenclature	Units	Description	Triple Hook	Conformal Cooling in Sliders
$\phi_{c}$	mm	Center channel diameter	9	-
$\phi_{i}$	mm	Side channel diameter	3	-
α		Horizontal separation angle between channels	120	-
β		Vertical slope of the channels	4.5	-
$\phi_{s}$	mm	Diameter conformal cooling channels in sliders	-	8
s	mm	Distance channel center—sliders surface	-	16

**Table 2:** Magnitude of the main properties of the material PC Lexan 121R.

Nomenclature	Units	Description	PC Lexan 121R
ρ_p_	g/cm^3^	Density	1.2
C_p_	J/kg·°C	Specific heat	1250
δ_p_	W/m·°C	Thermal conductivity coefficient	0.2
MFI	g/10 min	Melt flow index	17.5
T_mold_	°C	Mold Temperature (normal)	40
T_eject_	°C	Ejection temperature	100
T_freeze_	°C	Freeze temperature	164
E_p_	MPa	Elastic Modulus	2340
υ_p_	-	Poisson’s ratio	0.4
CLTE	1/°C	Coefficient of linear thermal expansion	6.84 × 10^−5^
UOI	-	Un-oriented refractive index	1.56
FSC	cm^2^/dyne	Flow-induced stress-optical coefficient	1.95 × 10^−10^
TSC	cm^2^/dyne	Thermally-induced stress-optical coefficient	4.50 × 10^−12^

**Table 3:** Mesh statistics for the meshes analyzed in the present manuscript.

Description	Units	Standard	Conformal and Fastcool
Part mesh node count	-	295,339	287,416
Part mesh element count	-	779,946	731,735
Part mesh volume	cm^3^	77.63	70.70
Runner mesh node count	-	2892	2892
Runner mesh element count	-	2464	2464
Runner mesh volume	cm^3^	0.05	0.05
Plastic part precisión (ε)—Mesh sizing	mm	1.00	1.00
Element type	-	Tetrahedral (10 nodes)	Tetrahedral (10 nodes)
Element type—Boundary layers	-	Prism (15 nodes)	Prism (15 nodes)
Offset ratioBoundary layers	-	0.1	0.1

**Table 4:** Magnitude of the physical properties of the defined materials.

Description	Units	Water Pure	PC Lexan 121R	Steel Alloy P20	Fastcool 50
Density	kg/m^3^	988	1200	7750	7810
Specific heat	J/kg·°C	4180	1250	460	470
Thermal conductivity coefficient	W/m·°C	0.643	0.200	29	50

**Table 5:** Magnitude of the physical properties of the defined materials.

Description	Units	Lexan 121R
Material type	-	Polycarbonate
Viscosity model	-	Modified Cross Model
PVT model	-	Modified Tait Model
Mechanical model	-	Isotropic pure polymer
Viscoelastic model	-	White-Metzner
Melt temperature	°C	280.0–310.0
Mold temperature	°C	70.0–95.0
Ejection temperature	°C	147
Freeze temperature	°C	164

**Table 6:** Magnitude of the physical properties of the defined materials.

Description	Units	Study Cases—Lexan 121 R (PC)
Filling time	s	2.21
Packing time	s	15.00
Cooling time	s	90
Melt temperatue	°C	295
Mold temperature	°C	80
Coolant temperature	°C	80
Maximum injection pressure	MPa	160
Maximum packing pressure	MPa	160
Packing pressure	MPa	128

**Table 7:** Time to reach ejection temperature for the analyzed cooling systems.

Cooling System Typology	Time to Reach Ejection Temperature [s]	Time Reduction [s]	Improvement [%]
Traditional	41.550	-	-
Conformal and fastcool	28.187	13.363	32.161

**Table 8:** Heat flux (J/s·cm^2^) for the analyzed cooling systems.

Cooling System Typology	Cavity Cooling	Core Cooling	Fastcool Insert	Total	Improvement [%]
Traditional	0.361	0.411	-	0.772	-
New conformal and Fastcool	0.501	0.903	1.430	2.834	267.10

**Table 9:** Cooling mold temperature difference for the analyzed cooling systems.

Cooling System Typology	Cooling Mold Temperature Difference (°C)	Temperature Reduction (°C)	Improvement (%)
Traditional	10.177	-	-
New conformal and Fastcool	1.557	8.620	84.701

**Table 10:** Warpage total displacement for the analyzed cooling systems.

Cooling System Typology	Warpage Total Displacement (mm)	Displacement Reduction (mm)	Improvement (%)
Traditional	0.875	-	-
New conformal and fastcool	0.557	0.318	36.343

**Table 11:** Warpage Von—Mises stress for the analyzed cooling systems.

Cooling System Typology	Warpage Von—Mises Stress (MPa)	Stress Reduction (MPa)	Improvement (%)
Traditional	5.319	-	-
Conformal and fastcool	1.634	3.685	69.280

## Data Availability

All data included in this study are available upon request by contact with the corresponding author.
